# Remotely Assessing Mechanisms of Behavioral Change in Community Substance Use Disorder Treatment to Facilitate Measurement-Informed Care: Pilot Longitudinal Questionnaire Study

**DOI:** 10.2196/42376

**Published:** 2022-11-07

**Authors:** Kevin A Hallgren

**Affiliations:** 1 Department of Psychiatry and Behavioral Sciences University of Washington Seattle, WA United States

**Keywords:** addiction, clinical pilot, measurement-based care, mechanisms of change, mobile health, mHealth, mobile phone

## Abstract

**Background:**

Research shows that improvements in coping strategies, abstinence self-efficacy, craving, and depression are potential mechanisms of behavioral change (MOBC) in treatments for substance use disorders (SUDs). However, little is known about how these insights regarding MOBC can be applied to SUD treatment settings. One way to facilitate MOBC-informed care in frontline settings could be to measure and monitor changes in MOBC throughout treatment using brief, frequent questionnaires that patients complete by using mobile technologies (eg, smartphones). The results derived from these questionnaires could potentially be used for clinical monitoring (ie, measurement-based care) to better understand whether individual patients are experiencing treatment-related improvements on key clinical targets.

**Objective:**

This study evaluated whether brief, weekly MOBC questionnaires completed by patients remotely can potentially provide clinically meaningful information about changes in MOBC in the context of real-world, community-based SUD treatment.

**Methods:**

A total of 30 patients (14/30, 47% female; 13/30, 43% racial or ethnic minority) in a community SUD treatment clinic participated in a pilot study where they were invited to complete brief, weekly questionnaires that assessed various MOBC, including coping strategies, abstinence self-efficacy, craving, depression, and therapeutic alliance. Questionnaires were typically completed remotely via smartphone for up to 6 months; 618 questionnaires were completed in total. Participants also completed longer, psychometrically validated measures of the same MOBC at baseline and 6-month research appointments. Statistical analyses tested whether brief, weekly, remotely completed MOBC questionnaires exhibited characteristics that would be desirable for real-world longitudinal clinical monitoring, including a tendency to detect within-person changes in MOBC over time; cross-sectional and longitudinal associations with longer, psychometrically validated measures completed at research appointments; and similar patterns of associations with 6-month percentage of days abstinent as longer, psychometrically validated MOBC measures completed at research appointments.

**Results:**

The results of this study indicated that the brief, weekly, remotely completed MOBC measures exhibited characteristics that are desirable for clinical monitoring, including a tendency to vary longitudinally (within patients over time) more often than measures of alcohol and drug consumption, generally having medium to large cross-sectional and longitudinal correlations with longer psychometrically validated measures of MOBC completed at research appointments, and generally having similar patterns of association with 6-month percentage of days abstinent from alcohol and drugs as longer psychometrically validated MOBC measures completed at research appointments.

**Conclusions:**

The results of this pilot study provide initial evidence that incorporating brief, weekly, and remotely completed MOBC questionnaires into community SUD treatment may be a viable approach for facilitating MOBC-informed care. Such questionnaires can potentially support measurement-based care by providing meaningful information about within-patient changes in clinical domains that are often directly targeted in SUD treatments and predict long-term substance use outcomes.

## Introduction

At least 2 million people receive treatment for substance use disorders (SUDs) annually in the United States [1]. Research has identified several efficacious treatments for SUDs, including behavioral and medication-based treatments [2,3]. To help improve the effectiveness and efficiency of SUD treatments, research has become increasingly focused on identifying the specific mechanisms of behavior change (MOBC) that may be most responsible for driving treatment outcomes [4,5].

Studies show that multiple MOBC—including (but not limited to) increases in substance-related coping skills and abstinence self-efficacy, reductions in craving and depression symptoms, and a strong therapeutic alliance—typically improve during a variety of SUD treatments, and these improvements often predict long-term substance use outcomes [6-19]. Moreover, these MOBC often reflect highly meaningful clinical targets for patients and clinicians; for example, patients may feel distressed by cravings and depression symptoms and often want to improve their coping skills and abstinence self-efficacy.

What remains less understood is how the growing body of MOBC-related findings will be used to improve the treatments that are offered in community-based SUD treatment settings; that is, how can MOBC science be used to help frontline clinicians offer better treatment? Because MOBC are potentially observable indicators of whether SUD treatments are working for individual patients in treatment, it is possible that measuring and monitoring MOBC longitudinally throughout treatment could help clinicians and patients detect whether a treatment is affecting meaningful proximal treatment targets that are associated with long-term outcomes (ie, MOBC). For example, clinicians could routinely administer brief questionnaires to assess different MOBC and then score, graph, and review changes in these measures over time throughout treatment as a form of treatment progress monitoring or measurement-based care [20]. These self-monitoring data could help draw clinical attention toward MOBC and facilitate clinical discussions about factors that may be contributing to changes in MOBC; for example, how specific behavior changes helped a patient increase their abstinence self-efficacy or reduce their depression symptoms. However, many community treatment settings are currently unable to systematically measure and monitor MOBC as a part of routine care. As a result, there are significant limitations to the extent that MOBC-related research findings affect the SUD treatments that patients receive in real-world settings.

To help reduce this gap, we developed a tool designed to facilitate MOBC-informed measurement-based care and pilot-tested its feasibility and usability in a community-based treatment setting [21,22]. The measurement-based care system included a patient-facing brief questionnaire that patients could complete remotely (eg, via smartphone), called *the weekly check-in*, which included questions assessing MOBC and other domains that clinicians reported as potentially helpful to measure frequently during routine care. The system also included a web-based dashboard for clinicians to review the longitudinal results of their patients’ weekly check-ins. During the clinical pilot, clinicians and patients had high rates of engagement with the system and provided favorable subjective ratings regarding its usability and clinical helpfulness [21].

Despite the high rates of engagement and favorable usability ratings of the weekly check-in [21], it is currently unclear whether brief and remotely completed weekly MOBC assessments could provide quantitatively meaningful data about changes in MOBC during real-world SUD treatment. Therefore, this study provides a preliminary evaluation of such MOBC assessments. If the results of this study indicate that such questionnaires provide quantitatively meaningful information, it would suggest that incorporating brief, remote, and frequent MOBC assessments during community SUD treatment could be a viable approach to facilitating MOBC-informed measurement-based care in real-world settings, which could be further tested in large-scale implementation studies. Specifically, this study evaluates whether brief, weekly, remotely completed MOBC questionnaires completed by patients in community-based treatment exhibited characteristics that would be desirable for real-world longitudinal clinical monitoring, including (1) a tendency to detect longitudinal within-person changes in MOBC over time; (2) cross-sectional and longitudinal associations with longer, psychometrically validated measures completed at research appointments; and (3) similar patterns of associations with 6-month percentage of days abstinent as longer, psychometrically validated MOBC measures completed at research appointments. It was hypothesized that the brief and remotely completed MOBC measures completed on the weekly check-in would vary longitudinally for most patients and that within-patient variability would be more common for measures of MOBC than for measures of alcohol and drug consumption, in part because many patients initiate abstinence from alcohol and drugs before starting SUD treatment [23,24]. It was also hypothesized that the remotely completed MOBC measures would have high cross-sectional and longitudinal associations with longer, psychometrically validated measures completed at baseline and 6-month research appointments, and that MOBC measures completed in both modalities—brief, remotely completed measures on the weekly check-in and longer, psychometrically validated measures completed at research appointments—would have similar patterns of association with 6-month percentage of days abstinent.

## Methods

### Setting and Participants for Clinical Pilot

In total, 30 participants were recruited from an addiction and mental health treatment clinic in an urban setting in Washington State, United States. The clinic offered treatment in various formats, including counseling (individual and group), peer recovery support, case management, pharmacotherapy for psychiatric, alcohol, and opioid use disorders, and harm reduction–oriented treatment (ie, for patients with nonabstinent goals). Participants were recruited via handouts that clinicians could give to patients during treatment sessions and flyers that were posted in public spaces within the clinic. Eligibility criteria for the clinical pilot included being enrolled in SUD treatment, receiving care from a clinician on 1 of 2 participating treatment teams, having an iPhone or Android smartphone, the ability to read and speak English, being at least 18 years old, and having an Alcohol Use Disorders Identification Test-Consumption version score >4 for men or 3 for women [25] or self-reporting past-year use of nonprescribed or illicit drugs, including alcohol, cannabis, hallucinogens, inhalants, opioids, sedatives, hypnotics, anxiolytics, or stimulants [26]. Eligibility screening was completed by phone by a member of the research team. Eligible patients were invited to complete a baseline appointment, where they provided informed consent to participate in the pilot. Participants completed additional follow-up research appointments with a research coordinator at 6, 12, and 24 weeks after baseline. Participants were recruited between October 2019 and June 2021. A sample size of 30 patients was recruited during this period, which was determined to be adequate for the formative aims of this research. Additional details regarding the setting and procedures are provided in a previous study [21].

### Measures

#### Weekly Check-in

A brief questionnaire, called *the weekly check-in*, was sent to patients every week via SMS text message or email through a REDCap server [27]. Patients completed the first weekly check-in at the baseline research appointment, and subsequent weekly check-ins were completed independently for up to 24 weeks. The weekly check-in contained 2 questions that assessed past-week substance use and 10 questions that assessed 5 MOBC domains described subsequently, with 1 to 3 questions per domain included from previously validated measures. The 5 MOBC domains were selected based on clinician input during formative research [22] and empirical evidence supporting their role as MOBC in SUD treatment.

The weekly check-in also included 10 other questions that were not evaluated here but were included in the weekly check-in based on clinician input [22]; these included 2 questions assessing positive life outlook, 6 questions assessing goals for the coming week, and 2 open-ended questions where patients could write additional goals or provide other notes they wanted to communicate to their clinicians. The specific questions included in the weekly check-in are provided in Multimedia Appendix 1 [28-32].

#### Research Assessments

Participants were also invited to complete longer, psychometrically validated measures of substance use and MOBC at research appointments completed at the baseline and at the 6-, 12-, and 24-week follow-ups. These appointments initially occurred in person but were later completed as telephone-based appointments to facilitate social distancing during the COVID-19 pandemic.

#### Substance Use and MOBC Domains

##### Overview

The measures used to assess substance use and MOBC domains are described in the following sections. Each section describes the brief, remotely completed measures that were included in the weekly check-in and the corresponding longer, psychometrically validated measures that were completed at the baseline and at the 6-, 12-, and 24-week research appointments.

##### Substance Use

On the weekly check-in, drinking and drug use were measured using 2 questions that asked participants how many days they “drank too much” and “used drugs” over the past week. The response options for both questions were on a 5-point scale ranging from “not at all” to “every day.” The 2 questions used in the weekly check-in were derived from the Substance Use Recovery Evaluator [32] with minor modifications to question wording based on earlier usability testing with patients that indicated some confusion with the original question phrasing [33].

At research appointments, drinking and drug use were assessed over a 30-day period via structured interviews with the Addiction Severity Index-Lite [34]. The specific indices used in this study reflected (1) the percentage of days (out of the past 30) that participants drank to intoxication, (2) the percentage of days (out of the past 30) that participants used any illicit or nonprescribed psychoactive drugs, excluding alcohol and tobacco, and (3) the percentage of days (out of the past 30) that participants reported complete abstinence from alcohol and illicit or nonprescribed psychoactive drugs, excluding tobacco.

##### Coping Strategies

The weekly check-in included 3 questions asking patients how often they used coping strategies that could be helpful for preventing alcohol and drug use. The 3 items were derived from the Coping Strategies Scale [28] and were selected for the weekly check-in because they reflected broad cognitive behavioral strategies that are applicable to a range of scenarios, including stimulus control (ie, avoiding people, places, and things that can lead to substance use), alternative reinforcement (ie, engaging in activities that can replace substance use), and high-risk planning (eg, planning ahead for situations that pose a high-risk for substance use). The wording of the questions and responses was modified to make the question structure more consistent with other items in the weekly check-in, as informed by preliminary usability testing with patients. Response options were on a 5-point scale ranging from “not at all” to “always.”

At research appointments, coping strategies were assessed using the 59-item Coping Strategies Scale [28], modified to reflect coping strategies relevant to preventing any type of substance use rather than alcohol specifically. The 59-item Coping Strategies Scale was initially excluded from the research appointment questionnaire battery owing to concerns about potential assessment fatigue to participants, as the questionnaire was considerably longer than all other measures. However, most patients were able to complete the initial questionnaire battery without assessment fatigue, so the questionnaire was added to the battery midway through the study. Therefore, it was only available at baseline for the last 14 enrolled patients and at the 6-month follow-up for the last 16 enrolled patients, one of whom declined to complete it because of assessment fatigue.

##### Abstinence Self-efficacy

The weekly check-in included two items asking patients how confident they felt in their ability to not drink or use drugs (ie, abstinence self-efficacy), including (1) when they were emotionally upset or in physical pain and (2) when they felt an urge or craving. The questions were derived from the Brief Situational Confidence Questionnaire-8 [29] with modifications to make phrasing more consistent with other items in the weekly check-in, as informed by preliminary usability testing with patients. Response options were on a 5-point scale ranging from “not at all confident” to “extremely confident.”

At research appointments, confidence in avoiding alcohol or drug use was assessed using the full Brief Situational Confidence Questionnaire-8 questionnaire [29].

##### Craving

The weekly check-in included one question that asked how many days the patient experienced alcohol and drug cravings over the past week using a question included in the Substance Use Recovery Evaluator [32]. Response options were on a 5-point scale ranging from “not at all” to “every day.”

At research appointments, craving was assessed over the past week using the 5-item Penn Alcohol Craving Scale questionnaire [35], which was modified to assess craving for drugs in addition to alcohol.

##### Depression

The weekly check-in included the Patient Health Questionnaire-2 (PHQ-2) to briefly assess depression symptoms [30]. The PHQ-2 is a 2-item measure derived from the first 2 items of the 9-item PHQ [36]. Response options are on a 4-point scale ranging from “not at all” to “nearly every day.”

At the research appointments, depression symptoms were measured using the full PHQ-9 [36].

##### Therapeutic Alliance

Therapeutic alliance was assessed on the weekly check-in using 2 questions asking patients to report how much their clinicians agreed with them about what was important to work on in treatment and how often their clinicians gave them new ways of looking at their problems. The 2 questions were derived from the Working Alliance Inventory-Short Revised [31] with modifications to make the questions and responses more consistent with the other questions on the weekly check-in. Response options were on a 5-point scale ranging from “seldom” to “always.”

At research appointments, the therapeutic alliance was assessed using the full Working Alliance Inventory-Short Revised [31].

### Ethical Considerations

All procedures used in this study were approved by the University of Washington Institutional Review Board (approval number: STUDY00007996). All participants provided written documentation of informed consent before participating in the study. All study data were deidentified before analysis. Research participants were paid US $50 in prepaid debit cards for attending each research appointment (up to 4 appointments or US $200 in total).

### Analysis Plan

Statistical analyses aimed to evaluate whether the MOBC measures obtained from the brief, remotely completed weekly check-in conveyed information that would be desirable for longitudinal monitoring of changes in MOBC during SUD treatment, including (1) a tendency to vary longitudinally within persons over time; (2) high cross-sectional and longitudinal associations with longer, psychometrically validated measures completed at research appointments; and (3) similar patterns of association with end-of-pilot percentage of days abstinent as the longer, psychometrically validated MOBC measures completed at research appointments.

Longitudinal, within-patient variability in weekly check-in domains was characterized descriptively to understand how much information each domain on the weekly check-in could potentially provide about *changes in* MOBC within patients over time (ie, measures with limited variability within patients over time may have less utility for longitudinal clinical monitoring and measurement-based care). Descriptive analyses were performed using data visualization methods that illustrated the variability and ranges of scores for weekly substance use and MOBC measures within patients across all time points. The number of patients whose weekly check-in responses indicated no variability over the 24-week period (eg, number of patients reporting no changes in substance use or no changes in coping strategies) was also identified to evaluate how often the weekly measures had no within-person variability.

Cross-sectional and longitudinal associations between the weekly check-in measures (assessed using 1-3 questions per domain) and their corresponding longer, psychometrically validated measures completed at research appointments (5-59 questions per domain) were examined. Cross-sectional associations were examined by computing Pearson correlations between the psychometrically validated measures and the corresponding weekly check-in measures when both were completed at baseline. Longitudinal associations were evaluated using Pearson correlations of *change scores* on the psychometrically validated measures between the baseline and the 6-month research assessments and *change scores* on the corresponding weekly check-in measures over a matched time period (ie, the “matched” weekly check-ins were those that were temporally closest to each research appointment, allowing a gap of up to 14 days between the research appointment and the weekly check-in).

Patterns of cross-sectional and longitudinal associations between percentages of days abstinent and MOBC domains assessed using both modalities (ie, weekly check-in and the longer, psychometrically validated measures completed at research appointments) were also examined. Cross-sectional analyses examined whether the pattern of Pearson correlations between the 6-month percentage of days abstinent and 6-month MOBC domains measured using both modalities were similar in magnitude and direction. Longitudinal analyses similarly examined whether the pattern of Pearson correlations between *changes in* percentage of days abstinent from baseline to 6 months and *changes in* MOBC over a matched period were similar in magnitude and direction for the MOBC measures obtained using both modalities.

Although the statistical power in Pearson correlation analyses was limited owing to the small sample size of this clinical pilot, the ability to test the aims of the study relied less on testing whether the correlation coefficients were statistically significant and more on examining whether the patterns of associations (effect sizes) observed for the weekly check-in measures were similar to the patterns of associations observed for the longer, psychometrically validated measures.

## Results

### Descriptive Statistics

A total of 30 patients were enrolled in this clinical pilot study. Of these, 8 (27%) patients were aged 18 to 34 years, 19 (63%) patients were aged 35 to 54 years, and 3 patients (10%) were aged ≥55 years. Furthermore, 53% (16/30) of the patients were male; 57% (17/30) of the patients were White, 13% (4/30) were Black, 7% (2/30) were American Indian or Alaska Native, 7% (2/30) were Hispanic or Latinx, and 17% (5/30) were from other racial or ethnic groups. The sample demographics were similar to those of the clinic’s patient population. Of the 30 patients, 90% (27/30) of the patients reported that they were not employed, and approximately half (16/30, 53%) reported that they were unhoused, had transitional or temporary or other housing, or were living in a house that someone else owned or leased. Patients self-reported that they were receiving treatment for the use of stimulants (18/30, 60%), opioids (16/30, 53%), alcohol (15/30, 50%), cannabis (5/30, 17%), sedatives (4/30, 13%), and hallucinogens (1/30, 3%). Additional patient descriptive statistics are available in a previous study [21].

Only 1 patient elected to receive the weekly check-in reminder via email; the remaining 29 patients received it via an SMS text message. Patients completed a mean of 20.60 weekly check-ins (SD 5.54; 85.8% of the maximum 24 weekly check-ins available to each patient; range 4-24; total number of weekly check-ins=618). Internal timing mechanisms on the weekly check-in indicated that, on average, the weekly check-in took <5 minutes to complete. All participants (30/30, 100%) completed the baseline research appointments. All but 1 patient (29/30, 97%) completed the 24-week research appointment. The participant who did not complete the 24-week research appointment also did not complete the 6- or 12-week appointments and was excluded from the analyses involving change scores.

### Within-Patient Variability in Weekly Substance Use and MOBC

**Figure 1:**
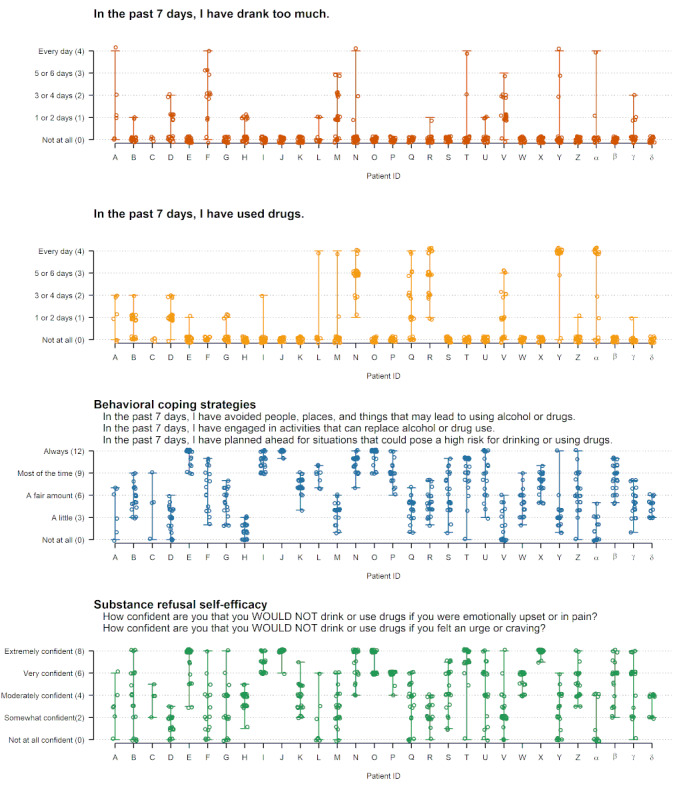
Within-patient variability in weekly check-in domains reflecting alcohol use, drug use, coping strategies, and substance refusal self-efficacy. Higher scores indicate more alcohol use, drug use, coping strategy use, and abstinence self-efficacy. Each letter on the x-axis reflects an individual patient and each dot reflects a score from a single weekly check-in. The vertical lines reflect each patient’s range of scores across repeated weekly check-ins.

**Figure 2:**
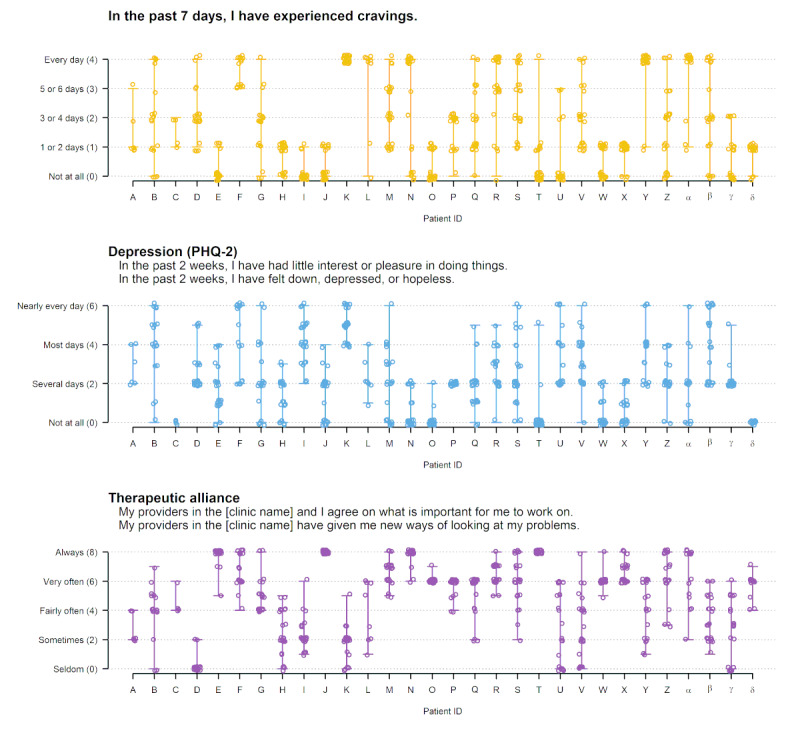
Within-patient variability in weekly check-in domains reflecting craving, depression, and therapeutic alliance. Higher scores indicate more frequent craving, higher self-efficacy, and higher therapeutic alliance. Each letter on the x-axis reflects an individual patient and each dot reflects a score from a single weekly check-in. The vertical lines reflect each patient’s range of scores across repeated weekly check-ins. PHQ-2: Patient Health Questionnaire-2

Within-patient variability over the 24 weeks of completing the weekly check-in is depicted in Figures 1 and 2. Each graph shows a separate weekly check-in domain, with each of the 30 patients represented by a different letter on the x-axis. Each weekly check-in score is represented as a dot, and the ranges of the scores reported for each patient are represented as vertical lines. For example, on the “drank too much” domain (Figure 1, top panel), patient A provided responses ranging from “not at all” to “every day” over the 24-week period (higher within-patient variability), patient B provided responses ranging from “not at all” to “1 or 2 days” (lower within-patient variability), and patient C only provided responses of “not at all” (no within-patient variability). As shown in Figure 1, a total of 50% (15/30) of the patients reported no variability on the drinking domain, 47% (14/30) of the patients reported no variability on the drug use domain, and 33% (10/30) of the patients reported no variability in both the drinking and drug use domains. In contrast, all patients displayed at least some variability in MOBC domains reflecting craving, coping strategies, and abstinence self-efficacy; 90% (27/30) of patients displayed at least some variability in depression and 93% (28/30) of patients displayed at least some variability in therapeutic alliance.

### Associations Between Weekly Check-in and Longer Psychometrically Validated Instruments

Scores on the first weekly check-in had significant cross-sectional correlations with scores obtained from longer, psychometrically validated instruments completed at baseline research appointments, with large effect sizes for domains reflecting substance use (*r*=0.51-0.64) and MOBC (*r*=0.57-0.85; Table 1). Correlations between *changes in* the weekly check-in measures and *changes in* the corresponding longer, psychometrically validated measures from baseline to 6 months were also significant, with large effect sizes (*r*=0.53-0.68) for domains reflecting *changes in* alcohol use, drug use, craving, depression, and abstinence self-efficacy. Correlations were significant, positive, and medium in size for *changes in* therapeutic alliance (*r*=0.42) but nonsignificant and smaller for *changes in* coping strategies (*r*=0.33).

**Table 1:** Correlations of substance use and mechanisms of behavioral change (MOBC) domains measured on brief, remotely completed weekly check-ins and substance use and MOBC domains measured on longer, psychometrically validated instruments completed at research appointments.

Weekly check-in domain		Cross-sectional correlations between domains measured on the weekly check-in and corresponding domains measured at research appointments at baseline			Longitudinal correlations between *changes in* domains measured on the weekly check-in and *changes in* corresponding domains measured at research appointments, from baseline to 6 months		
		*r* (95% CI)	Number of observations included in the analysis	*P* value	*r* (95% CI)	Number of observations included in the analysis	*P* value
**Substance use**							
	Alcohol use	0.51 (0.19 to 0.74)	30	.004	0.63 (0.33 to 0.82)	27	<.001
	Drug use	0.64 (0.37 to 0.82)	30	<.001	0.53 (0.19 to 0.75)	28	.004
**MOBC**							
	Coping strategies^a^	0.57 (0.02 to 0.85)	13	.04	0.33 (−0.30 to 0.76)	12	.29
	Abstinence self-efficacy	0.73 (0.51 to 0.87)	30	<.001	0.62 (0.33 to 0.81)	28	<.001
	Craving	0.81 (0.63 to 0.91)	30	<.001	0.57 (0.24 to 0.79)	26	.002
	Depression	0.85 (0.70 to 0.93)	30	<.001	0.68 (0.42 to 0.84)	28	<.001
	Therapeutic alliance	0.72 (0.48 to 0.86)	30	<.001	0.42 (0.06 to 0.69)	28	.03

^a^A standardized questionnaire for coping strategies was only added to the research appointment assessment battery midway through the study because of initial concerns about potential assessment fatigue to patients (ie, the Coping Strategies Scale contains 59 items). Therefore, it was only available at baseline for the last 14 enrolled patients and at the 6-month follow-up for the last 16 enrolled patients, one of whom declined to complete it because of assessment fatigue at the research appointment.

### Associations Between MOBC Measures and 6-Month Percentage of Days Abstinent

The 6-month percentage of days abstinent generally had similar patterns of association with MOBC measured via the weekly check-in and MOBC measured using longer, psychometrically validated measures at research appointments. In cross-sectional analyses at 6 months (Table 2: cross-sectional correlation between MOBC measure and percentage of days abstinent, both measured at 6 months), effect sizes of the associations between percentages of days abstinent and MOBC were similar in magnitude and direction for MOBC measured via the weekly check-in versus MOBC measured via longer, psychometrically validated measures completed at research appointments (ie, absolute differences in Pearson correlation effect sizes, *r*, were always <0.10) for coping strategies, abstinence self-efficacy, depression, and therapeutic alliance. However, for craving, the cross-sectional effect size was larger in magnitude for the modified Penn Alcohol Craving Scale completed at the research appointments compared with the single-item craving measure completed remotely on the weekly check-in. Although detecting statistical significance was not a primary objective of this analysis, 6-month percentages of days abstinent had significant cross-sectional associations with 6-month coping strategies measured on the weekly check-in, 6-month abstinence self-efficacy measured on the weekly check-in and at research appointments, and 6-month craving measured at research appointments.

For the analyses of change scores (Table 2: longitudinal correlation between changes in MOBC measure and changes in percentage of days abstinent, both examined from baseline to 6 months), the effect sizes of the associations between *changes in* percentages of days abstinent and *changes in* MOBC domains were similar when MOBC were measured via the weekly check-in and when MOBC were measured via longer, psychometrically validated measures completed at research appointments for all domains (ie, absolute difference in Pearson correlation effect size, *r*, was always <0.10). Although detecting statistical significance was not a primary objective of this analysis, *changes in* the percentages of days abstinent were significantly associated with *changes in* coping strategies measured on the weekly check-in and *changes in* therapeutic alliance measured on the weekly check-in and at research appointments.

**Table 2:** Correlations between percentages of days abstinent and mechanisms of behavioral change (MOBC) domains measured on brief, remotely completed weekly check-ins and MOBC domains measured on longer, psychometrically validated instruments completed at research appointments.

MOBC domain		Cross-sectional correlation between MOBC measure and percentage of days abstinent, both measured at 6 months				Longitudinal correlation between *changes in* MOBC measure and *changes in* percentage of days abstinent, both examined from baseline to 6 months		
		*r* (95% CI)	Number of observations included in the analysis	*P* value	*r* (95% CI)		Number of observations included in the analysis	*P* value
**Coping strategies**								
	Weekly check-in	0.45 (0.09 to 0.70)	28	.02	0.42 (0.05 to 0.68)		28	.03
	Research appointments^a^	0.40 (−0.14 to 0.76)	15	.14	0.36 (−0.27 to 0.77)		12	.25
**Abstinence self-efficacy**								
	Weekly check-in	0.62 (0.33 to 0.81)	28	<.001	0.26 (−0.13 to 0.57)		28	.19
	Research appointments	0.58 (0.27 to 0.79)	28	.001	0.26 (−0.12 to 0.58)		28	.18
**Craving**								
	Weekly check-in	−0.25 (−0.57 to 0.13)	28	.19	−0.29 (−0.60 to 0.10)		27	.14
	Research appointments	−0.58 (−0.78 to −0.25)	27	.002	−0.37 (−0.66 to 0.01)		27	.06
**Depression**								
	Weekly check-in	−0.02 (−0.39 to 0.36)	28	.94	−0.26 (−0.58 to 0.12)		28	.18
	Research appointments	−0.11 (−0.46 to 0.28)	28	.58	−0.29 (−0.60 to 0.09)		28	.13
**Therapeutic alliance**								
	Weekly check-in	0.26 (−0.13 to 0.58)	28	.18	0.49 (0.14 to 0.73)^a^		28	.008
	Research appointments	0.31 (−0.07 to 0.61)	28	.11	0.54 (0.21 to 0.76)^a^		28	.003

^a^A standardized questionnaire for coping strategies was only added to the research appointment assessment battery midway through the study because of initial concerns about potential assessment fatigue to patients (ie, the Coping Strategies Scale contains 59 items). Therefore, it was only available at baseline for the last 14 enrolled patients and at the 6-month follow-up for the last 16 enrolled patients, one of whom declined to complete the study due to assessment fatigue.

## Discussion

### Principal Findings

This study provided a preliminary evaluation of whether brief, weekly, remotely completed, patient-reported MOBC assessments could provide quantitatively meaningful information about changes in MOBC during community-based SUD treatment. The results of this pilot study provide preliminary support for our hypotheses; the weekly MOBC measures (1) varied considerably within patients over time and typically varied more than measures of substance use; (2) had large cross-sectional and longitudinal associations with longer, psychometrically validated MOBC measures completed at research appointments; and (3) had patterns of cross-sectional and longitudinal associations with percentages of days abstinent that were generally similar in magnitude and direction as longer, psychometrically validated MOBC measures completed at research appointments. The results of this pilot study provide promising preliminary support for the feasibility of remotely measuring multiple MOBC domains via a brief weekly patient self-report questionnaire for providing clinically meaningful information during SUD treatment as usual as it is offered in a community treatment setting.

There were 2 findings that did not align with the study’s hypotheses; one involved the 3-item coping strategies measure, which had a small longitudinal association with the 59-item Coping Strategies Scale despite there being a high cross-sectional association between these 2 measures and similar patterns of association with the percentage of days abstinent. One potential reason for this discrepancy is that the Coping Strategies Scale’s assessment of 59 specific cognitive behavioral coping strategies may capture more nuanced changes in coping behavior because it captures 59 specific behavioral changes that patients could make, in contrast to the 3 coping strategies measured on the weekly check-in that were intended to capture more general coping strategies that are applicable for a range of situations. Nonetheless, the similar cross-sectional and longitudinal associations of both coping measures with percentages of days abstinent suggest that both may capture important information about coping strategies that may help monitor patients’ use of strategies that can help with avoiding substance use. The second finding that was inconsistent with the hypotheses involved the weekly check-in measure of craving, which had a nominally smaller cross-sectional association with the percentage of days abstinent at 6 months than the 5-item Penn Alcohol Craving Scale. A broader assessment of craving across multiple dimensions (eg, frequency and intensity) using the Penn Alcohol Craving Scale may provide a more complete picture of patients’ experiences with craving, particularly as craving relates to current substance use. However, notably, the single-item craving measure on the weekly check-in had large cross-sectional and longitudinal associations with the Penn Alcohol Craving Scale, suggesting that a single-item question on the weekly check-in may capture much of the same information as the Penn Alcohol Craving Scale, potentially warranting the use of a single-item craving measure when the benefits of a briefer assessment outweigh the potential costs of a longer measure (eg, if a 5-item measure is potentially burdensome to patients or impractical for a given clinical setting).

Previous studies have shown that abstinence self-efficacy, depression symptoms, and craving can be reliably measured using brief questionnaires [30,37,38]. Previous work has also shown that a brief, clinician-administered assessment measure that includes questions about MOBC can predict substance use outcomes when administered every 3 months in the context of an effectiveness trial [39]. This study builds on these findings and shows that brief, weekly, remotely completed, patient-reported MOBC assessments can provide quantitatively meaningful information about changes in MOBC when embedded in a measurement-based care system that is added to community-based SUD treatment. Measuring MOBC briefly, remotely, and using patient self-reports may increase the feasibility of longitudinal, multidimensional measurement-based care in SUD treatment. The resulting information may help clinicians monitor multiple dimensions that could help indicate whether treatment is affecting the MOBC, which are expected to improve during evidence-based treatments and predict long-term substance use outcomes. Monitoring in such a manner could help clinicians obtain frequent information to support measurement-based care and could complement less frequent clinician-administered assessments [39] and patients’ narrative reports about their treatment progress.

Monitoring MOBC could also potentially help guide clinical attention toward the importance of MOBC as pertinent treatment targets; for example, by reminding patients that treatment can address coping strategies, self-efficacy, craving, and depression symptoms—not just alcohol and drug consumption [40,41]. The results from this study suggest that there may be a particular utility in measuring hypothesized MOBC in SUD treatment rather than focusing measurement specifically on alcohol and drug *consumption*, given the tendency for MOBC to vary over time more often than measures of substance use. Measures of *substance use* are typically specified as primary treatment outcome measures in SUD clinical trials [42,43] and are among the most common outcomes for patients and clinicians to focus their attention on during SUD treatment; for example, clinicians often check in with their patients whether they are currently using substances, how often they are using them, or how long they have gone without using substances. In contrast, MOBC (eg, coping strategies, abstinence self-efficacy, craving, depression symptoms, and therapeutic alliance) may be discussed less frequently in routine care despite representing highly pertinent domains that can cause distress (eg, craving, depression), motivate treatment-seeking (eg, low self-efficacy or limited coping strategies), signal risk for treatment dropout (eg, low therapeutic alliance [44]), and are often directly addressable with interventions that clinicians can offer during sessions (eg, practicing new coping skills, helping patients obtain medications for craving or depression, and clarifying reasons for poor therapeutic alliance).

### Limitations and Strengths

This study had several limitations. Because this was a pilot study, the sample was intentionally small, which limited the statistical power to detect statistical significance in some correlational analyses. In addition, the 59-item Coping Strategies Scale was only added to the assessment batteries for the last 14 enrolled patients owing to concerns about it creating assessment fatigue; therefore, the statistical power was further limited for analyses involving this measure. Despite the small sample size, some associations were found to be statistically significant. Moreover, the overarching goal of this pilot study was to compare patterns of association between the weekly check-in and longer, psychometrically validated measures rather than to test whether any given association was statistically significant. This study was conducted in a single urban addiction and mental health clinic, and future studies are needed to evaluate the performance of tools that briefly and longitudinally assess MOBC in additional community-based treatment settings, including those with varying service models, workflows, and patient populations. The brief questionnaires used in this study were not previously validated, and this study lacked an adequate sample size to perform analyses that focused on psychometric validation. However, the questions were derived from longer, validated measures, and this study is still able to show proof-of-concept that measuring MOBC using brief, weekly, remotely completed, patient-report questionnaires can potentially provide clinically meaningful information.

This study also had noteworthy strengths. The remote monitoring system tested here was developed with multiple rounds of patient and clinician inputs. It was tested as an add-on to SUD treatment as it is usually offered in a community setting, which provides high external validity, and it was found to be engaging, usable, and clinically helpful by patients and clinicians [21]. Thus, the tested system may be feasible to incorporate into other community settings. Follow-up rates were high. Despite the smaller sample size for the pilot study, the frequency of measurement yielded a large number of data points (over 600 weekly check-ins completed, over 20 repeated measures per patient on average). The sample was also diverse in terms of gender and race.

### Conclusions

Measuring MOBC frequently and remotely using brief patient-report measures may be a viable approach for obtaining clinically meaningful information about changes in MOBC during treatment. Implementing systems that facilitate measurement and monitoring MOBC longitudinally during SUD treatment could be one approach to support the delivery of MOBC-informed care in community treatment settings.

## Appendix

Multimedia Appendix 1 Questions included in the weekly check-in.

## Abbreviations

MOBC: mechanisms of behavioral change
PHQ: Patient Health Questionnaire
SUD: substance use disorder

## Data Availability

The data sets generated during this study are not publicly available because consent was not obtained from participants to share the research data publicly; however, the data are available from the corresponding author upon reasonable request.
