# Supplementary material for: Remotely Assessing Mechanisms of Behavioral Change in Community Substance Use Disorder Treatment to Facilitate Measurement-Informed Care: Pilot Longitudinal Questionnaire Study
Source: JMIR Form Res. 2022 Nov 7;6(11):e42376. doi: 10.2196/42376 (PMC9679949; doi:10.2196/42376)
Supplement: Multimedia Appendix 1 [file formative_v6i11e42376_app1.docx]

**Multimedia Appendix 1**. Questions included in the weekly check-in.

**The weekly check-in**

The questions included on the weekly check-in are provided below. The questions were derived from existing measures, including the Substance Use Recovery Evaluator (Neale et al., 2016), the Patient Health Questionnaire-2 (Kroenke et al., 2003), Coping Strategies Scale (Litt et al., 2003), Brief Situational Confidence Questionnaire-8 (BSCQ-8; Breslin et al., 2000), and Working Alliance Inventory-Short Revised (WAI-SR; Munder et al., 2010).
